# Supplementary material for: Understanding the role of exosomal lncRNAs in rheumatic diseases: a review
Source: PeerJ. 2023 Dec 13;11:e16434. doi: 10.7717/peerj.16434 (PMC10725171; doi:10.7717/peerj.16434)
Supplement: Supplemental Information 1 [file peerj-11-16434-s001.docx]

Take the PubMed search engine for example:

1.(("Exosomes"[Mesh]) OR ("Exosomes*")) AND (("RNA, Long Noncoding"[Mesh]) OR ("RNA, Long Noncoding*"))) AND (("Arthritis, Rheumatoid"[Mesh]) OR ("Arthritis, Rheumatoid*"))

2.((("Exosomes"[Mesh]) OR ("Exosomes*")) AND (("RNA, Long Noncoding"[Mesh]) OR ("RNA, Long Noncoding*"))) AND (("Osteoarthritis"[Mesh]) OR ("Osteoarthritis*"))

3.((("Exosomes"[Mesh]) OR ("Exosomes*")) AND (("RNA, Long Noncoding"[Mesh]) OR ("RNA, Long Noncoding*"))) AND (("Lupus Erythematosus, Systemic"[Mesh]) OR ("Lupus Erythematosus, Systemic*"))

4.((("Exosomes"[Mesh]) OR ("Exosomes*")) AND (("RNA, Long Noncoding"[Mesh]) OR ("RNA, Long Noncoding*"))) AND (("Liver Cirrhosis, Biliary"[Mesh]) OR ("Liver Cirrhosis, Biliary*"))

5.((("Exosomes"[Mesh]) OR ("Exosomes*")) AND (("RNA, Long Noncoding"[Mesh]) OR ("RNA, Long Noncoding*"))) AND (("Dermatomyositis"[Mesh]) OR ("Dermatomyositis*"))

6.((("Exosomes"[Mesh]) OR ("Exosomes*")) AND (("RNA, Long Noncoding"[Mesh]) OR ("RNA, Long Noncoding*"))) AND (("Scleroderma, Systemic"[Mesh]) OR ("Scleroderma, Systemic*"))

7. 1 AND 2 AND 3 AND 4 AND 5 AND 6

Annotation: Pubmed allows truncated word searches using * as a wildcard.
